# PosiGene: automated and easy-to-use pipeline for genome-wide detection of positively selected genes

**DOI:** 10.1093/nar/gkx179

**Published:** 2017-03-15

**Authors:** Arne Sahm, Martin Bens, Matthias Platzer, Karol Szafranski

**Affiliations:** Leibniz Institute on Aging, Fritz Lipmann Institute, 07745 Jena, Germany

## Abstract

Many comparative genomics studies aim to find the genetic basis of species-specific phenotypic traits. A prevailing strategy is to search genome-wide for genes that evolved under positive selection based on the non-synonymous to synonymous substitution ratio. However, incongruent results largely due to high false positive rates indicate the need for standardization of quality criteria and software tools. Main challenges are the ortholog and isoform assignment, the high sensitivity of the statistical models to alignment errors and the imperative to parallelize large parts of the software. We developed the software tool PosiGene that (i) detects positively selected genes (PSGs) on genome-scale, (ii) allows analysis of specific evolutionary branches, (iii) can be used in arbitrary species contexts and (iv) offers visualization of the results for further manual validation and biological interpretation. We exemplify PosiGene's performance using simulated and real data. In the simulated data approach, we determined a false positive rate <1%. With real data, we found that 68.4% of the PSGs detected by PosiGene, were shared by at least one previous study that used the same set of species. PosiGene is a user-friendly, reliable tool for reproducible genome-wide identification of PSGs and freely available at https://github.com/gengit/PosiGene.

## INTRODUCTION

‘What is the genetic basis of phenotypic differences between species?’ is a recurring question in comparative genomics. A frequently used method is to search for genes that evolved under positive selection. Positive selection describes the phenomenon that beneficial gene variants become fixed in a population/species over time because they increase fitness. It is a major evolutionary mechanism that leads to fixation of innovation and adaptation to changing environmental conditions (1,2). Most commonly, the **ω** ratio (the non-synonymous to synonymous substitution rate ratio, also known as d_N_/d_S_ or K_a_/K_s_) is used as a sign for positive selection on protein-coding genes.

Systematic scans for positively selected genes (PSGs) have provided insights into adaptation processes. For example, PSGs were identified for many well known bacterial pathogens that have immune related counterparts on the mammalian side (3–7). A similar ‘arms race’ can be found between venomous animals and their predators or preys (8,9). Genome-scale searches linked PSGs to phenotypic traits like subterranean life and longevity of mole-rats (10–12), the ability of Tibetan antelopes to live in high altitudes with low oxygen-concentration (13) and increased mitochondrial efficiency leading to lower ROS-levels in ants as potential prerequisite for their remarkable long lifespan (14). Moreover, a significant role of positive selection on neuronal-expressed genes in the evolution of the human nervous system was illustrated (15).

Despite important insights gained by many genome-wide works, re-evaluation studies have stated false-positive rates of predicted PSGs between 45 and 90% (16–19). As the respective original studies are based on locally developed and implemented computational tools, this led to heterogeneous quality standards, absence of reproducibility and eventually, to incongruent results (10,16,20).

There is a lack of a general software solution that offers automated and reliable analysis of genome-scale data. Several challenges are contributing to this situation. First, such a software solution must be applicable in a general way, which means that an ortholog assignment approach is required that allows arbitrary species sets to be used and consequently, arbitrary evolutionary branches to be tested. Second, the management of alternative splice variants is an important aspect in a eukaryotic context. Since the majority of eukaryotic genes are expressed as multiple transcripts it is necessary to select representative isoforms for further downstream analyzes. Choosing the longest isoform or picking at random can be a substantial source of false positives, because these approaches increase the chance of misalignments due to the inclusion of non-homologous regions, such as those derived from species-specific exons. Instead, isoforms should be chosen that are likely to be similar from a functional and evolutionary perspective – but also in a reasonable amount of time (21). Third, evolutionary codon models as the backbone of PSG identification are highly sensitive to bad quality of input data. Errors can originate from sequencing, assembly and gene annotation as well as pseudogenes that were not recognized as those. Furthermore, errors can occur during the different steps of the genome-wide PSG search itself, e.g., if gene fragments or poorly conserved sequences are assigned to an ortholog group. Another source of errors lies in applying the statistical models on alignments showing non-conserved regions that cannot be resolved without ambiguity. All these problems can lead to alignments of non-homologous codons resulting in a statistical signal that is misinterpreted as positive selection. On genome scale even low rates of false signals can outnumber the true candidates (16–18). This is why strict quality-filtering strategies are necessary to ensure reliable results (16,19). Fourth, it is imperative to efficiently parallelize large parts of the software, because most of the steps it has to conduct, like ortholog assignment, high quality multiple sequence alignment (MSA) and application of codon substitution models, have considerable computational costs. Execution of such steps on a single processor for thousands of genes is not practicable within a reasonable amount of time (22).

Genome-scale PSG searches require considerable experience in bioinformatics. To simplify the PSG search several attempts have been made over the recent years (Table 1). The tools Datamonkey (23), Selecton (24) and JCoDA (25) were developed to simplify the procedures for single-gene studies in particular steps: alignment of orthologous sequences, computation of the phylogenetic tree and/or configuration of tools that implement codon substitution models. IDEA (26) is a graphical program that allows to analyze multiple genes in parallel but requires pre-aligned sequence data and virtually lacks a filtering procedure or data quality control to ensure plausibility of the predicted candidates. PhyleasProg (27) and PSP (28) are able to perform all necessary steps for genome-wide PSG identification but are restricted to fix sets of few vertebrate species or bacteria strains, respectively. The recently developed end-to-end pipeline POTION (29) meets most of the requirements. However, it does not offer a solution for branch-specific PSG search, which is the common application scenario because it allows to link identified PSGs to phenotypic traits (1,10,14,15,30–35).

**Table 1.** Features of existing software in the field of PSG identification

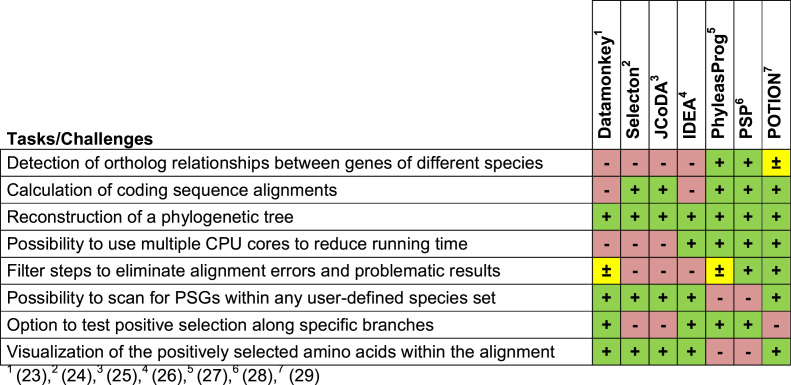

Toward user-friendly, reliable tools for reproducible genome-wide identification we developed PosiGene that addresses all the above mentioned challenges and performs the complex analysis automatically. In addition, PosiGene offers alignment visualization, in which positively selected protein sites and functional domains are highlighted. We validated PosiGene on simulated data using sequences with known features of positive selection and on real data comparing its results against those of five high-ranking publications on positive selection along the human lineage.

## MATERIALS AND METHODS

### Structure and workflow of the PosiGene pipeline

#### Overview

The minimal required input comprises coding sequences – in FASTA or GENBANK format – for all species to be analyzed. The output consists of a table showing all genes (including those that are not significant) ranked by their probability to be under positive selection and includes information about positively selected sites, d_N_/d_S_ ratios as well as links to alignment visualizations.

**Figure 1.**
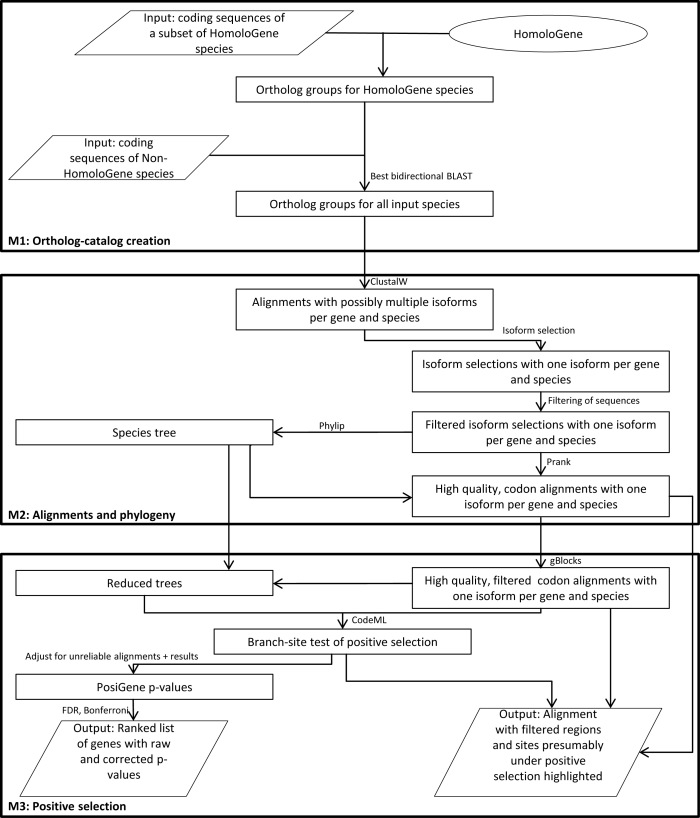
Workflow diagram of PosiGene.

A user manual (https://github.com/gengit/PosiGene/blob/master/doc/User_Guide.pdf) provides detailed information about all possible parameters that can be used to customize PosiGene. The software is divided in three consecutive modules: the first module (M1) builds the ortholog catalog, i.e. the genome-wide set of ortholog assignments, based on the user-defined set of species and sequences. The second module (M2) constructs alignments and derives a phylogenetic species tree. The third module (M3) scans genes for positive selection along a user-chosen branch of the species tree. PosiGene can be called in a way that all modules are executed consecutively or to run a single module separately. The latter feature can be used to add a species to the ortholog catalog, change parameters or to search another branch for PSGs without having to rerun the whole pipeline (Figure 1).

PosiGene is implemented in Perl and uses different Bioperl (36) modules for reading and writing sequence, tree as well as alignment files. All modules – except the HomoloGene based ortholog assignment at the beginning of M1 (see below), which stresses Input/Output – are heavily parallelized. Threads are created once at the beginning of each submodule (Figure 1) and are reused efficiently for new tasks by the main thread via queues. This avoids extra or inhomogeneous computational load caused by thread administration.

All arguments used by PosiGene to call incorporated third party programs are listed in Supplementary Table S1.

#### M1: building the ortholog catalog

The assignment of genes to ortholog groups is the basis of later analyses. We have implemented a mixture of core species with already established ortholog relations and automated orthology prediction for any user-supplied species’ data. This ensures reliability as well as flexibility of the ortholog assignment system.

Ortholog groups are determined, in a first approach, based on the HomoloGene database (37). The local HomoloGene copy is contained in the program package and currently contains 21 species covering a wide evolutionary range (http://www.ncbi.nlm.nih.gov/homologene/statistics/). Sequences of species that are not part of HomoloGene are assigned to the initial ortholog groups by a best-bidirectional BLAST hit criterion (38,39), which was adapted to resolve multiple isoforms per gene, using group-to-group instead of sequence-to-sequence assignment. We define group-to-group assignment such that a gene ***X*** of a species that is not part of HomoloGene is assigned to a homology group ***Y***, as defined by HomoloGene, if and only if the best hit across all isoforms of ***X*** is within ***Y*** and vice versa. The best-bidirectional hit criterion was shown to perform well in comparison with other ortholog assignment methods, irrespective of phylogenetic distance (40).

The module M1 is skipped if the user provides ortholog assignments of the sequences.

#### M2: alignments and phylogeny

The first step in this module is a similarity-based sequence selection to ensure that, per subsequently conducted positive selection test, there will be only one transcript isoform per species. Therefore, to each isoform of an ‘anchor species’ the most similar isoform of each other species is assigned. The anchor species of a PosiGene run is chosen by the user and could be, as a recommendation, the best annotated species with the most complete set of coding sequences or a species whose lineage shall be tested subsequently for positive selection. The isoforms that are most similar to the anchor species’ sequences are determined via an initial MSA on protein level calculated by CLUSTALW. For this all possible isoforms from each species in an ortholog group are used. In comparison to pure pairwise alignments, the progressive nature of CLUSTALW, which aligns more similar sequences first, decreases the chance of aligning non-homologous regions, such as alternative exons. In comparison to the subsequently used PRANK, the widely used aligner CLUSTALW is much faster and thus, be able to produce results on large, i.e. many sequence containing, MSAs in a feasible amount of time (41). This is important because many genes are spliced into multiple isoforms. Finally, there are as many isoform assignments per ortholog group as there are isoforms in the anchor species. Generally, all following procedures, including M3, will be applied to the obtained isoform assignments.

Next, highly divergent sequences are removed from the isoform assignments. Each non-anchor species sequence whose similarity with the anchor species sequence does not reach a threshold will be removed. Furthermore, in order to guarantee an adequate level of conservation between the non-anchor species sequences, each of them is required to fulfill a second similarity threshold, in respect to all other non-anchor species sequences. The latter rule is implemented by iteratively removing sequences, beginning with the sequence that violates the rule most often. If multiple sequences violate the rule with equal frequency, the sequence that has the lowest similarity to the anchor species sequence is removed first.

For subsequent analysis steps, a phylogenetic tree is needed. The user can either provide a species tree, or it will be computed from the previously calculated isoform assignments using the parsimony method of the PHYLIP package (42) and jackknifing. Briefly, for this step, those isoform assignments are used that contain, after aforementioned sequence filtering, still all species that were specified by the user at the beginning. The aligned isoform assignments are concatenated and then cut in chunks of equal length. Each chunk is filtered with GBLOCKS (43) to remove gaps and unreliable alignment columns, following a tree reconstruction based on the filtered chunks with DNAPARS of the PHYLIP package. Dnapars carries out unrooted parsimony (44) and uses the method of (45) to calculate branch lengths. From these trees a consensus tree is calculated with CONSENSE of the same package and unrooted afterward. Since CONSENSE does not predict consensus branch lengths, we calculate the average branch length for every node of the consensus tree over all nodes of the chunk trees that are equivalents of the respective consensus tree node.

All isoform assignments that comprise at least three sequences (which means also three species) are aligned now on codon level using PRANK (46). The choice of the alignment software has a large impact on the result of PSG identification (18,47). PRANK produces the most reliable candidates in this context, as was found on simulated as well as real data (18,19,48,49). As guide tree the species tree is used (see above).

#### M3: positive selection and filtering

To identify genes under positive selection on specific evolutionary branches, we use the PAML package (50,51). PAML is widely used as a framework to test phylogenetic hypotheses by using maximum likelihood based on estimation of the **ω** ratio. Specifically, we use the CODEML program of the PAML package to conduct the branch-site test of positive selection on each PRANK MSA (52,53). Briefly, this test is conducted by calculating and comparing the likelihoods of a null model, under which all sites may evolve under neutral or negative selection and an alternative model, under which the sites of the targeted branch are additionally allowed to evolve under positive selection. The *P*-value for the likelihood ratio test is calculated via a χ^2^ distribution with one degree of freedom. Besides a PRANK alignment, CODEML is supplied with a phylogenetic tree reduced to the species that are represented in the respective MSA, if necessary. Simulations have shown that the branch-site test has good accuracy and statistical power. However, it is sensitive to alignment as well as sequence errors and tends to produce more false negatives in scenarios of few, very similar or very short sequences due to low information content (54,55). Besides nominal *P*-values PosiGene results provide correction for multiple testing using the Bonferroni and Benjamini–Hochberg methods. Specific sites under positive selection are inferred by the Bayes emiprical Bayes method (56) implemented in CODEML.

**Figure 2.**
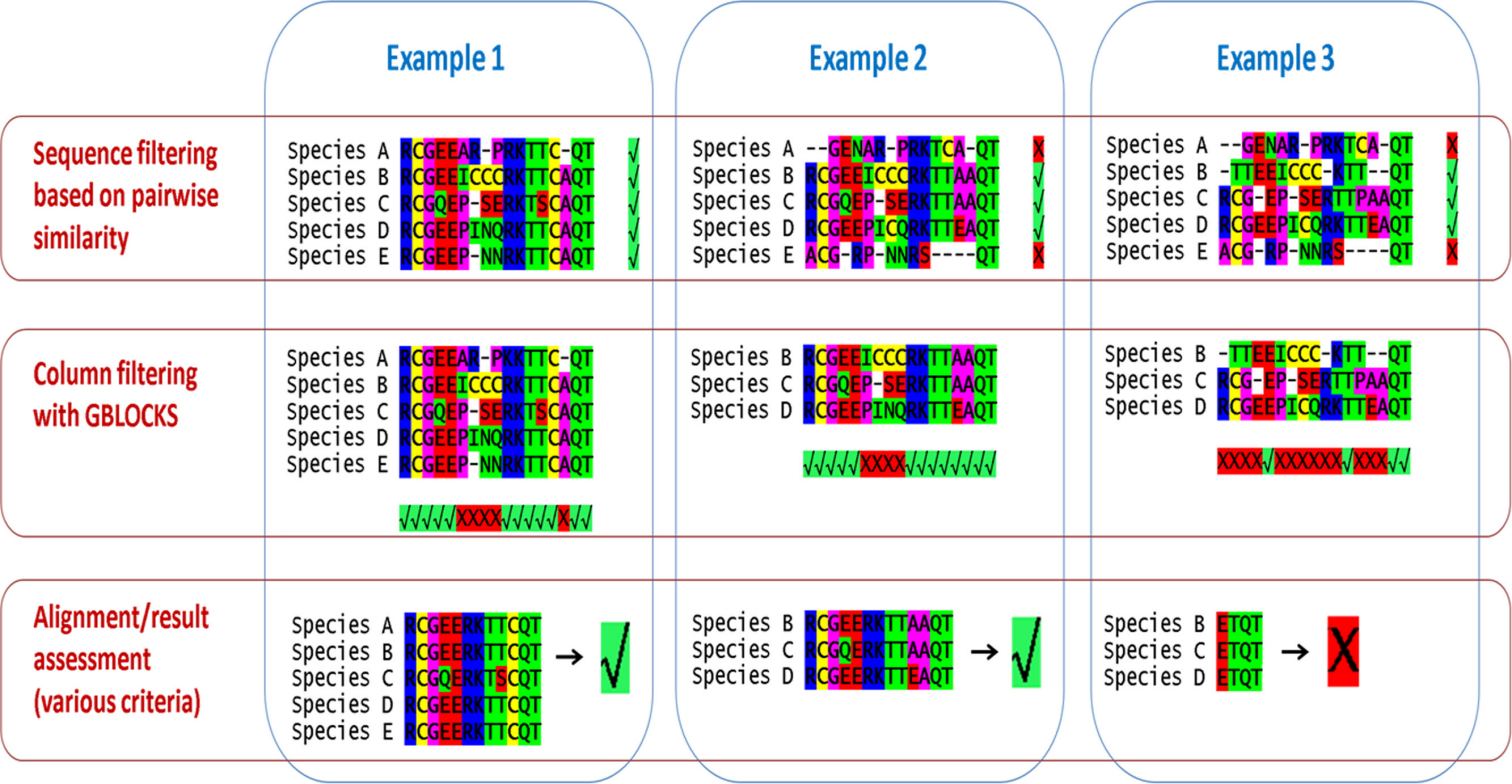
Schematic illustration of the filtering in PosiGene. Three approaches that are conducted at different steps of the program are depicted. The red marked ‘X’ means that the respective sequence/species, alignment column or the whole alignment/result were removed from further analysis, while the green marked √ means that the filter was passed. The shown examples are artificial and serve for demonstration only. In particular, for Example 3, the minimum length for a block of accepted alignment columns is depicted shorter (one codon/amino-acid) than in real application. The reason why the alignment in Example 3 does not pass the filter would be that a too small fraction of the alignment passed the column filter. For compact illustration, all steps are shown on protein level, while the column filtering works in reality on codon level.

As part of the PosiGene workflow, we paid special attention to minimize potential false positive PSGs by implementing a series of filtering steps (Figure 2). First, gaps and surrounding unconserved alignment columns are stringently removed with GBLOCKS (43) from the PRANK MSAs. A filtering of questionable alignment columns is necessary, because alignment of non-homologous codons is a major source of false positives (16). Second, as was mentioned, sequences failing pairwise similarity thresholds are deleted from alignments early in the workflow. MSAs containing those sequences are likely to have many disordered regions, promoting the alignment of non-homologous codons. This filtering step can also be seen as an instrument to reduce false negatives. Few badly conserved sequences can force the first mentioned filter to delete large parts of the MSA reducing the power of the test and potentially removing positively selected sites. Third, entire MSAs can be discarded if they are considered unreliable for the following reasons, if: (i) a small absolute number or a small percentage of alignment columns or anchor species codons remain after the first filtering step, (ii) few sequences remain after the second filtering step, (iii) disproportional d_N_/d_S_ ratios (e.g. ≥100 in foreground branch) were calculated by CODEML or (iv) an implausibly high fraction of positively selected sites was inferred. Additionally, MSAs will only be considered if at least one species from the sister taxon (i.e. the most closely-related species/clade) of the examined branch is represented in it. Without this condition it is not possible to say whether potentially observed selective pressure worked on the branch of interest or before in evolution (57).

The alignment visualization component processes four kinds of information: the MSA itself, the probability for each site to be under positive selection, which parts of the MSA were removed by GBLOCKS and thus could not be analyzed, as well as functional domains that are potentially listed in the GENBANK file of the anchor species. The information is depicted in two ways: first, as Portable Network Graphics (PNG) in different display formats based on Bioperl and the GD Graphics library; second, as a file type that is interpretable by Jalview (58). Jalview is a free Java based program for MSA visualization that is delivered with the PosiGene package and integrated insofar as PosiGene's Jalview visualizations can be opened with one simple command. Jalview also allows the user to edit the alignment, e.g., by adding further annotations.

### Validation methods

#### Valdiation on simulated data

First, we tested PosiGene based on simulated coding sequences that were generated with INDELible (59). Note, that the branch-site tests evaluates, for a given coding sequence, whether the assumption that a proportion of codons is target to positive selection on the tested branch fits the data significantly better than the assumption that all its codons evolved under neutral or purifying selection. Selective pressure is represented by the **ω** ratio and **ω** > 1 indicates positive selection.

**Figure 3.**
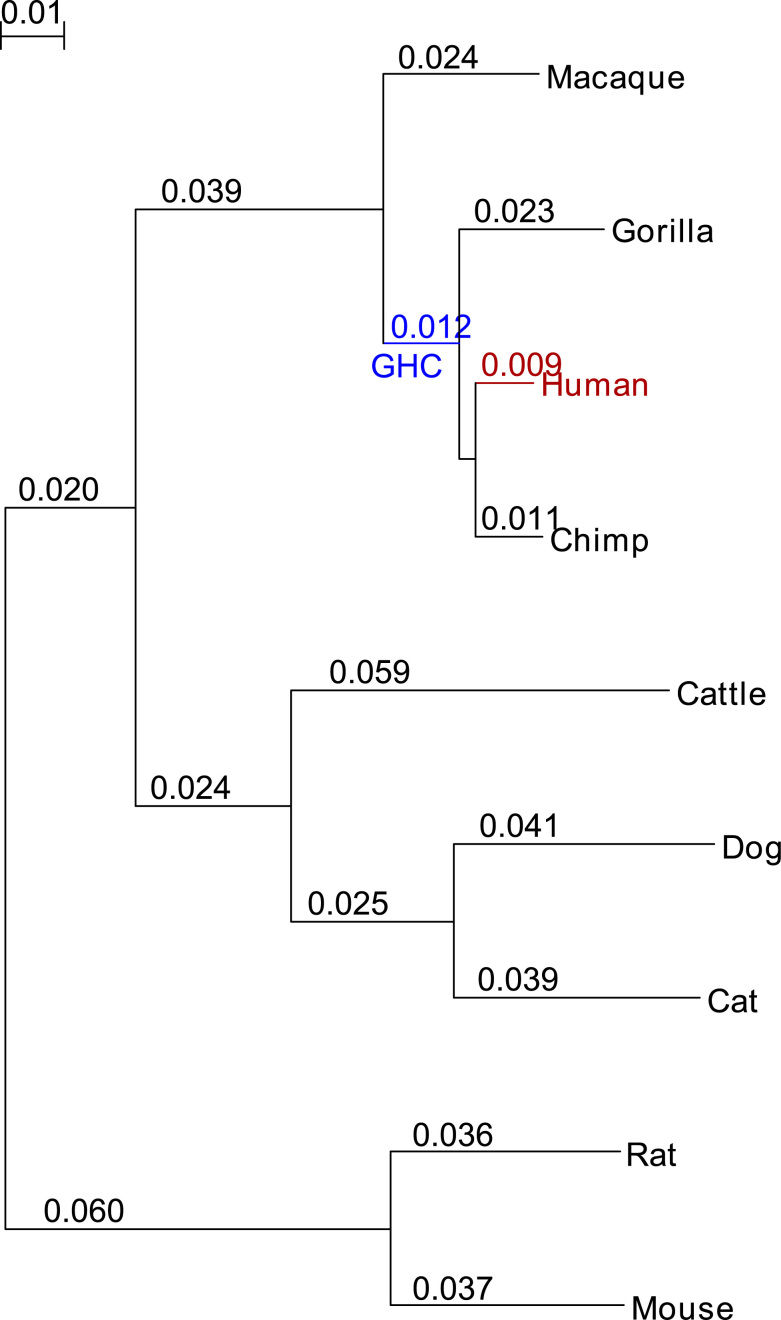
Species set used in real data validation. Shown is the phylogenetic tree that was calculated by PosiGene with the displayed species set. Branch lengths are drawn in scale and additionally shown directly at the branches. The respectively tested branches (both times human) are colored red. The tree was furthermore used to generate simulated sequences. In the simulation, the branches were tested that are equivalent to the red colored branch (human) and the blue colored branch (last common ancestor of GHC). In some of the simulation runs respectively one of these two branches was simulated to be under a different selection scheme than all other branches of the tree.

In order to assess the false positive rate we simulated the evolution of 1000 coding sequences by a selection scheme ***N*** without sites under positive selection. In scheme ***N***, the sitewise selective pressure was set to a discrete distribution that was previously estimated based on 6.05 million codons in 12 871 gene trees comprising 29 mammals (60). However, we replaced the 0.99–1.0 quantile (the only one with **ω** > 1) with the weighted average of all other quantiles **ω** = 0.21222 (Supplementary Table S2). Indels were modeled with a geometric length distribution with parameter *q* = 1−*p* = 0.35 resulting in a mean and standard deviation of 1.54 respectively 0.91 codons. This distribution, developed in a similar simulation study (48), adequately fits published data on coding sequences of mammalian genomes (61,62). We used a ratio of substitution to indels of 43 as it was found in coding regions of primates (62). The ratio of transition to transversion substitutions, **κ**, was fixed at 2 and the stationary codon frequency of α-globin from our real data validation was used. For a realistic test scenario the sequences were evolved along the phylogenetic tree of the real data validation. However, the branch lengths had to be multiplied with three in order to conform with a different concept of branch lengths used by INDELible. We verified that the branch lengths that were predicted by PosiGene on the simulated datasets match those of the original tree. All branches of the tree were simulated to evolve under selection scheme ***N*** (Supplementary Table S2). The root sequence length was set to 400. Finally, we configured PosiGene to search separately on one internal as well as on a terminal branch of the tree for PSGs to test the program for both possibilities. The tested terminal branch was the one that corresponds with the human branch in the real data validation (see Figure 3) and the internal branch corresponds with the last common ancestor of human, chimp and gorilla (GHC). Of note, both tested branches were simulated (as all others) to evolve under selection scheme ***N***, i.e. without positive selection.

In order to assess sensitivity we used in principle the same simulation model with the modification that the two tested branches now were evolved under selection schemes ***A–E***. The other branches were still simulated under selection scheme ***N*** as before. The schemes ***A–E*** differ from ***N*** insofar as a proportion of sites with **ω** > 1 was added. The signal for positive selection was concentrated on 1, 3, 5, 7 and 9% of the codons for the schemes ***A–E***, respectively (Supplementary Table S2). Its strength was adjusted to fit an overall average **ω** of 0.9 - indicating still for a moderate purifying selection over the whole sequence. For each scheme ***A–E*** and for each of the both tested branches again 1000 sequences were generated.

#### Validation on real data

To determine the congruency among the five human studies as well as POTION and PosiGene results, we converted all candidate IDs to Ensembl human gene IDs. Due to historical reasons, multiple Ensembl gene IDs can refer to the same gene. Therefore we performed a last translation step and took the Ensembl gene names as objects of comparison. In congruency with most of the regarded studies (15,30,34,35) we defined candidates by having passed the filters of the respective work and nominal *P*-values equal or below 0.05 based on the branch-site test of positive selection. For ID conversion (Supplementary Table S3) we used Ensembl Biomart (63), except for the conversion of UCSC transcript IDs used by (1) to RefSeq transcript IDs for which we used the UCSC Table Browser (64). Additionally, for the OrthoMCL (65) cluster names that are used in the POTION output we determined the human protein IDs within the respective cluster and used them for further conversion. PosiGene were run on two different species sets: one with four species and one with nine species. Since there is no gold standard for PSGs, we define true candidates as being identified by at least two (respectively at least three) of the examined studies. Thus, the precision of a given study is defined as following:

\begin{equation*}{\rm{precision}} = \frac{{\left| {\left\{ {{\rm{study\ candidates}}} \right\}{\rm{\ }}\mathop \cap \nolimits^ {\rm{\ }}\left\{ {{\rm{true\ candidates}}} \right\}} \right|}}{{\left| {\left\{ {{\rm{study\ candidates}}} \right\}} \right|}}\end{equation*}

### Benchmarking

For both PosiGene runs that were conducted in the frame of the real data validation, i.e. the four-species as well as the larger nine-species set, we measured how much total central processing unit (CPU) time was consumed and how much real time was needed to complete each of the three PosiGene modules (Table 2). For the benchmarking, we used a computer with 24 Intel Xeon processors of which each had a clock rate of 2.5 GHz. The differences between CPU time divided by the numbers of used processors and the real time that was needed, have to be mostly attributed to input/output operations on files. In the module M1 of the four-species run there is even less CPU time needed than real time due to the circumstance that all four-species were part of the HomoloGene database and thus no BLAST steps were performed (see M1: building the ortholog catalog). PosiGene's memory consumption is negligible.

**Table 2.** Real and CPU time needed to run PosiGene on the real datasets analyzed in this work

	4-species set		9-species set	
	Real time	CPU time	Real time	CPU time
**M1: building the ortholog catalog**	1.8 h	1.1 h	17.1 h	305.3 h
**M2: alignments and phylogeny**	6.6 h	125.1 h	26.8 h	565.6 h
**M3: positive selection and filtering**	5.1 h	95.7 h	33.9 h	799.5 h
**∑**	13.5 h	221.9 h	77.8 h	1670.4 h

Note: the table shows the real and CPU times consumed by two PosiGene runs that were executed on species sets of different sizes. A server with 24 processors was used for both runs.

## RESULTS AND DISCUSSION

**Figure 4.**
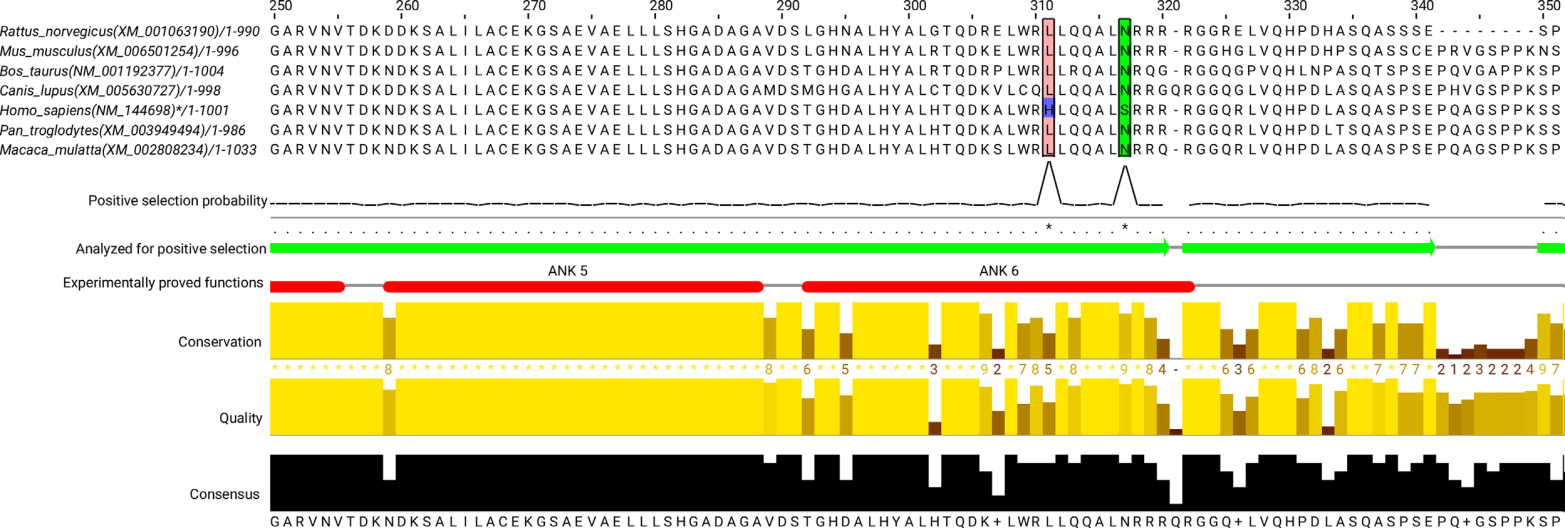
PSG visualization by PosiGene. Shown is a subregion of the ANKRD35 alignment. Human residues identified to be under positive selection (L311H, N317S) are colored with respect to physico-chemical properties using the Zappo code (http://www.jalview.org/help/html/colourSchemes/zappo.html). The probability of each residue to be positively selected is indicated by a line below the alignment and displayed upon a mouse-over action. Below are highlighted parts of the alignment that were used for the PSG test (green arrows) as well as experimentally supported protein domains based on an annotated sequence file (red bars). The three plots for conservation, quality and consensus at the bottom represent column-wise measures for the conservation of the physico-chemical properties of the amino-acids based on the Analysis of Multiply Aligned Sequences (AMAS) method (68), the likelihood of observing the mutations based on the BLOSUM62 matrix (69) and the percentage of the modal residue, respectively.

The newly developed end-to-end pipeline PosiGene is the first bioinformatics tool for the detection of PSG that performs the following analysis steps automatically: (i) determination of ortholog relationships between genes of different species, (ii) calculation of coding sequence alignments, (iii) reconstruction of a phylogenetic tree, (iv) filtering procedures for unreliable alignment data and implausible results as well as (v) the branch-site test of positive selection. Each step is heavily parallelized to reduce running time. PosiGene consists of three modules: M1 ortholog catalog creation, M2 alignments and phylogeny, M3 positive selection (Figure 1). It offers alignment visualization, in which positively selected protein sites and functional domains are highlighted. This enables biologists to manually validate and functionally interpret specific sites in individual candidates (Figure 4). Additionally, non-experts get an easy-to-use tool with reliable default parameters, while experts can configure the program to their needs and make use of its modularization. The PosiGene pipeline was applied successfully in several studies for genome-scale PSG identification (57,66,67). PosiGene is designed to run on linux platforms instantly without further installation and is available at https://github.com/gengit/PosiGene.

To validate PosiGene's performance we used simulated and real data.

### Validation on simulated data

First, we validated PosiGene on simulated coding sequences. The basic idea of this approach is to simulate the evolution of protein-coding sequences with defined selection schemes along the branches of a phylogenetic tree. This enabled us to create scenarios, in which PosiGene should detect positive selection (scenarios ***A–E***) and a scenario in which it should not (scenario ***N***). As tree we used the same as in the real data approach (Figure 3) and tested, in each scenario, the branches: (i) human, as a representative of a terminal branch or (ii) the last common ancestor of gorilla, human and chimp (GHC), as an internal branch (Table 3).

**Table 3.** PosiGene's performance on simulated gene trees

Scenario	Codons under selection	Tested branch	Identified PSGs^1^	Description
***N***	0%	Human	0.3%	False positive rates
		GHC^2^	0.4%	
***A***	1%	Human	30.3%	True positive rates^3^
		GHC	30.7%	
***B***	3%	Human	15.0%	
		GHC	15.0%	
***C***	5%	Human	8.6%	
		GHC	10.2%	
***D***	7%	Human	6.2%	
		GHC	7.4%	
***E***	9%	Human	5.4%	
		GHC	6.1%	

^1^A PSG was defined by having a nominal *P*-value ≤ 0.05.

^2^GHC – last common ancestor of gorilla, human and chimp.

^3^The overall strength of positive selection was identical for scenarios *A–E* (ω = 0.9) resulting in highest concentration of the selection pressure in scenario *A* and lowest in *E*.

PosiGene results (p≤0.05) of scenario ***N*** indicate false positive rates of 0.3 and 0.4% in the human and the GHC branch, respectively. The true positive rates, determined in scenarios ***A–E***, lie between 5.4 and 30.7%, Supplementary Figure S1 shows false and true positive rates depending on how the *P*-value threshold is chosen. In order to assess PosiGene's false and true positive rates, we compared them with values from previously published extensive simulation experiments (48). In this study, Fletcher and Yang reported for the branch-site test of positive selection false positive rates without filtering between 2.1 and 13.0%. If, as only filtering procedure, gaps were removed from the alignment the false positive rates were between 2.4 and 10.2%. If alignment methods other than PRANK were used, the false positive rates were even higher. So, with a rate of 0.3–0.4% PosiGene's filtering of the alignments efficiently suppresses false positives. This, however, raises the question of whether our strict filtering procedures diminish PosiGene's true positive rate? This would be the case if the filtering removes true alignment signals. Since in simulations the ‘true alignments’ are known, Fletcher and Yang used these alignments directly to assess the maximum true positive rate that can technically be achieved using the branch-site test, and obtained rates between 1.4 and 33.1% (48). The fact that PosiGene's true positive rates (5.4–30.7%) were within the upper range of these estimates indicates that the negative impact of its filtering procedures is low. In regard to the still relatively high number of false negatives produced by the branch-site test, it should be noted that the coding sequences were simulated with an overall average signal of moderate negative selection and only a small fraction of codons were allowed to evolve under positive selection.

Furthermore, we observe that PosiGene's sensitivity is positively correlated with the concentration of the signal of positive selection (Pearson correlation; *r*^2^ = 0.89, *P*-value 0.04), i.e. PosiGene's ability to detect positive selection increases if few sites are affected by heavy selective pressure (scenario ***A***) and decreases if many sites are influenced by weak selective pressure (scenarios ***B–E***).

### Validation on real data

While simulations offer the advantage of precise knowledge about the selective pressures that influenced sequence evolution, they may not cover the full range of problems that occur in analysis of real genome-wide data, e.g. the existence of paralogs, which currently cannot be simulated as above. However, since there is a lack of an independent validation technique for real data PSG candidates, we could not define a single study as a gold standard. Instead, we used the agreement between different studies as an indication for precision (positive predictive value) of predictions. Previous works have pinpointed precision in favor of sensitivity as major goal of PSG analysis on genome scale (16–18,20).

For the real data validation approach, human served as a useful lineage because it has been analyzed multiple times for PSGs on a genome wide scale. Therefore, we took the PSG candidates from five human studies (1,15,31,33–35). In addition, we compared PosiGene only to the recently developed end-to-end pipeline POTION due to the principal limitations of other existing tools (Table 1). We ran POTION with default settings and complete mRNA sequence sets from human, chimp, mouse, rat, dog and maquaque as input (Supplementary Table S4). This species set is reduced in comparison to the set that was given to PosiGene due to the limitations of the OrthoMCL-based ortholog assignment system used by POTION, which restricts easy, semi-automatic ortholog assignment to species that are present in the OrthoMCL database (65). The species additionally used for the PosiGene run were cattle, cat and gorilla (Figure 3). To test the effect of the size of the used species set, an independent PosiGene run with only four species was conducted: human, chimp, maquaque and mouse. The PSG candidates of both PosiGene runs (Supplementary Tables S5 and 6) were predicted with default settings and human was set to be the tested species. Details about the examined studies like used alignment software, species set and filtering mechanisms are summarized in Supplementary Table S7.

We measured consensus on two levels: PSGs that were found by at least one, respectively, two other studies (Table 4, Supplementary Tables S8 and 9). The study of Clark *et al*. (33) shows least consistency with the other works. Since it is the earliest work, this could be explained by fewer and less qualitative gene sequences, availability of only two species for comparison to the tested human branch and use of an older version of the branch-site test that was improved subsequently (52,53). Also the POTION pipeline produced small intersections with the other works. However, this performance is hardly comparable because POTION uses site tests, which check whether a gene was generally under positive selection during evolution, instead of the branch-site tests performed by the other works. The scope of application cases given with the presentation of POTION suggests that the program's default parameters, especially the filtering parameters, were optimized for PSG analysis in bacterial or less complex eukaryote genomes (29). Finally, we remark that the works of Kosiol (1) and Bakewell (34) show the best results in terms of sensitivity, that is, the absolute number of predicted PSGs confirmed by other studies.

**Table 4.** Congruency of human PSG predictions across different studies with PosiGene nine-species result

Study	Found PSGs	Shared by at least one other study		Shared by at least two other studies	
		Absolute	Precision [%]	Absolute	Precision [%]
Clark, *et al.* (33)	525	22	4.2	9	1.7
Arbiza, *et al*. (35)	146	61	41.8	41	28.1
Bakewell, *et al.* (34)	138	88	63.8	56	40.6
Kosiol, *et al.* (1)	204	103	50.5	59	29.0
Gaya-Vidal and Alba (15)	190	65	34.2	43	22.7
POTION	123	8	6.5	5	4.1
PosiGene	98	67	68.4	47	48.0

On both measured consensus levels, PosiGene has consistently the highest precision, with more than two-third and almost the half of genes that were found by at least one, respectively, two other studies. This outperformance is not explained by the size of the species set used for branch-site analysis. A reduction of the species set from nine to four results in even a slightly increased precision, regarding PSGs that are shared by at least one other study and only in a minimal drop of precision from 48.0 to 44.7%, regarding PSGs that shared by at least two other studies (Supplementary Table S10). While the reduction of the species set does not negatively affect precision it does reduce sensitivity: the number of identified PSGs drops from 98 to 47. However, this is expected due to the decreased power of the branch-site test in alignments with fewer sequences (54). We acknowledge that, within the comparison, PosiGene identifies the fewest PSG candidates, potentially indicating a high false negative rate. This could be attributed to the circumstance that we laid our focus on precision instead of sensitivity, in agreement with the literature (16–18,20). In respect to co-supported candidates, however, only the Bakewell and Kosiol studies (1,34) identified more PSGs showing that PosiGene's sensitivity can compete with that of the other studies. Of note, the fully automated pipeline of PosiGene is compared against the primary results of high ranking studies, which were able to use tailored data quality controls that are difficult to implement in a generally applicable program. For example, the Bakewell study, which has the highest precision besides of PosiGene, integrated the nucleotide qualities of the chimpanzee genome as a main filtering mechanism into their approach. Furthermore, the studies neither had the aim nor provided tools to reproduce their approach. Arbiza, Bakewell and Gaya-Vidal (15,34,35) also did not provide the alignments which further hinders evaluation of the results and follow-up studies. In contrast, PosiGene offers the possibility of easy reproduction of results that were predicted by others and provides alignment visualizations to manually verify, biologically interpret and experimentally examine PSGs and selected sites.

## CONCLUSIONS

The identification of PSGs is a prevailing genomics approach that enabled insights into adaptation processes, molecular function and the genetic source of species-specific phenotypic traits. PosiGene can be used with a single command line call to search for relevant candidates on a user-chosen evolutionary branch and a genome-wide scale. Besides a list of genes that are ranked according to the probability to be under positive selection, PosiGene generates alignment visualizations which enable to contextually interpret the positively selected amino acid sites within the respective candidate.

We compared the functionality of PosiGene with other tools that partly enable to search for PSGs on different scales. We argue that none of them would be suited as a broadly applicable tool for genome-wide searches that aim to link phenotypic traits of a species or clade to its PSGs, because important aspects like filtering mechanisms, a freely selectable species set or a branch-specific analysis are lacking.

We demonstrated PosiGene's performance in two complementary validation strategies. One validation was based on simulated data giving precise control over targets of positive selection. It was shown that PosiGene's filter mechanisms result in a very small false positive rate that is a fraction of known values for unfiltered data. Since simulated data may not cover the full range of possible problems, a second validation on real data was performed. The results demonstrated that PosiGene reaches a good overlap with existing high-ranking studies on the human lineage, e.g., more than two-third of the PSGs that were identified by PosiGene were also found by at least one human study.

Altogether, we provide PosiGene as step toward a user-friendly tool for genome-wide identification of PSGs that produces reliable results reproducible by others which can be visualized for further manual validation and biological interpretation.

## AVAILABILITY

Project name: PosiGene

Project home page: https://github.com/gengit/PosiGene

Operating System: linux 64-Bit

Programming language: perl

License: GPL Version 3

## Supplementary Material

Supplementary Data
