# Supplementary material for: PosiGene: automated and easy-to-use pipeline for genome-wide detection of positively selected genes
Source: Nucleic Acids Res. 2017 Mar 15;45(11):e100. doi: 10.1093/nar/gkx179 (PMC5499814; doi:10.1093/nar/gkx179)
Supplement: Supplementary Data [file gkx179_supp.zip › nar-03686-met-n-2016-File006.pdf]

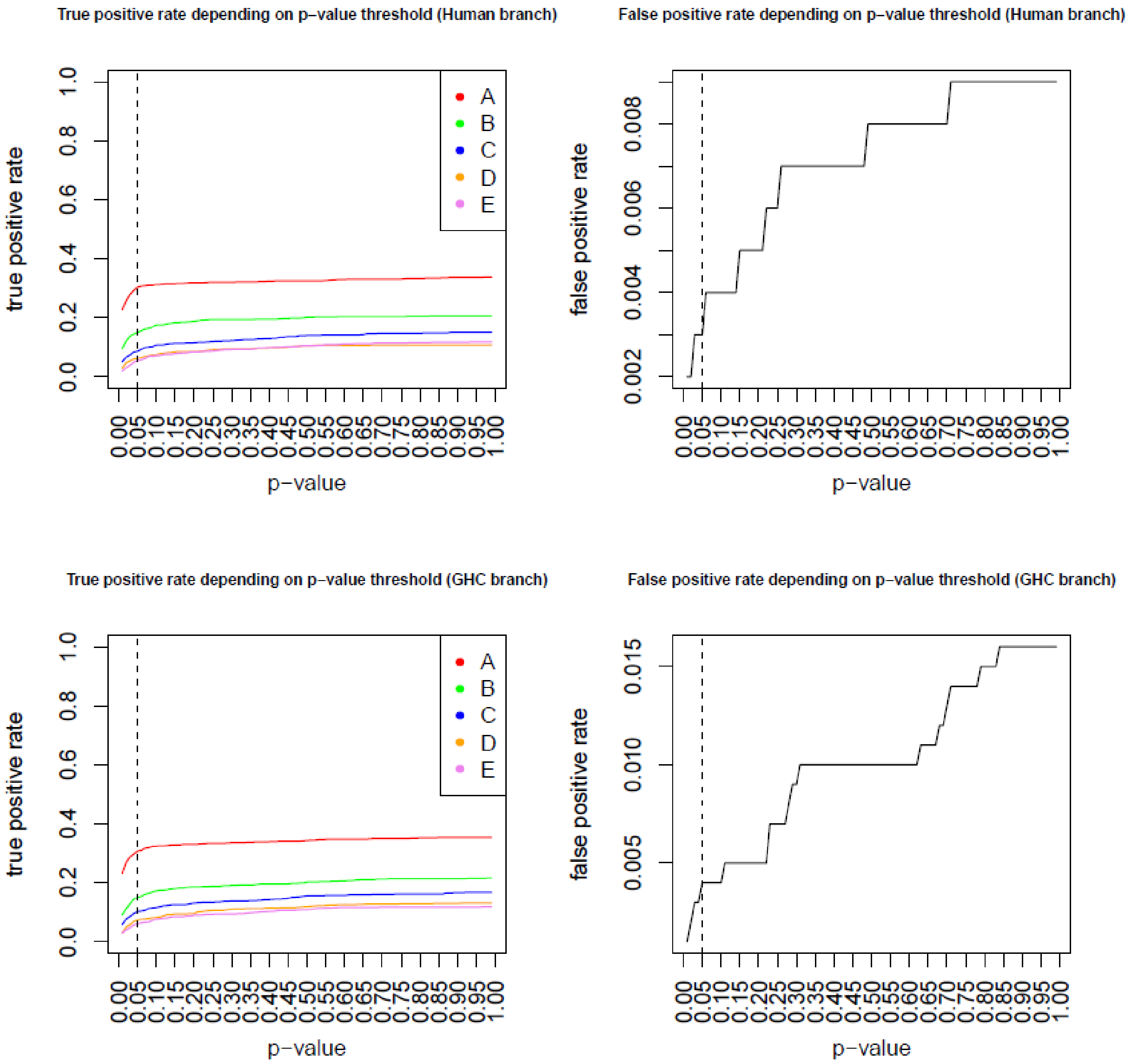

Figure S1. True and false positive rates depending on p-value threshold. Shown are the dependencies of the true positive rates (left) and false positive rates (right) on the p-value that is used as significance threshold. The dashed line marks the p-value threshold of 0.05 that was used in the article. The different colored curves in the true positive plots represent the different selection scenarios (*A*-*E*) that were described in the article.
