# Supplementary material for: PosiGene: automated and easy-to-use pipeline for genome-wide detection of positively selected genes
Source: Nucleic Acids Res. 2017 Mar 15;45(11):e100. doi: 10.1093/nar/gkx179 (PMC5499814; doi:10.1093/nar/gkx179)
Supplement: Supplementary Data [file gkx179_supp.zip › nar-03686-met-n-2016-File007.docx]

**Table S1. Overview of software used by PosiGene.**

**Table S2. The ω-distributions that were used in computer simulations.**

**Table S3. ID-conversion in real data comparisons.**

**Table S4. Positively selected genes identified by POTION.**

**Table S5. Positively selected genes identified by PosiGene with 9 species.**

**Table S6. Positively selected genes identified by PosiGene with 4 species.**

**Table S7. Methodical overview of compared studies.**

**Table S8. Pairwise overlaps of positively selected genes between studies.**

**Table S9. Positively selected genes identified by different studies.**

**Table S10. Congruency of human PSG predictions across different studies with PosiGene 4-species result.**
